# Temperature Measurement during Abrasive Water Jet Machining (AWJM)

**DOI:** 10.3390/ma15207082

**Published:** 2022-10-12

**Authors:** Damian Bańkowski, Piotr Młynarczyk, Irena M. Hlaváčová

**Affiliations:** 1 Department of Metal Science and Manufacturing Processes, Faculty of Mechatronics and Mechanical Engineering, Kielce University of Technology, al. Tysiąclecia Państwa Polskiego 7, 25-314 Kielce, Poland; 2 Department of Physics, Faculty of Electrical Engineering and Computer Science, VSB–Technical University of Ostrava, 17. Listopadu 2172/15, Ostrava, 70800 Poruba, Czech Republic

**Keywords:** cutting temperature, abrasive water jet, temperature measurement, XRD analysis, jet impact zone, computed tomography inspection

## Abstract

This study was undertaken to look for confirmation that heat transfer induced by abrasive water jet machining (AWJM) affects the microstructure of the material cut. The structure of S235JR carbon steel used in the experiments was reported to change locally in the jet impact zone due to the high concentration of energy generated during cutting with the abrasive water jet. It is assumed that some of the energy is transferred into the material in the form of heat. This is particularly true for materials of considerable thickness with a high thermal conductivity coefficient when cutting is performed at low speeds or with high abrasive consumption. The literature on the subject suggests that in AWJM there is little or no thermal energy effect on the microstructure of the material cut. The research described here involved the measurement of the cutting temperature with thermocouples placed at four different distances from the edge. The distances were measured using computed tomography inspection. The thermocouples used in the tests were capable of detecting temperatures of up to 100 °C. Locally, temperatures at the edge may reach much higher values. The results of the X-ray diffraction qualitative phase analysis reveal that locally the temperatures may be much higher than the eutectoid temperature. Phase changes occurred along the edge since austenite was observed. This suggests that the temperature in the jet impact zone was much higher than the eutectoid temperature. Optical microscopy was also employed to study the material microstructure. Finally, the material nanohardness was determined.

## 1. Introduction

Abrasive water jet machining is known to be a complex high energy process [1,2,3]. The large amounts of kinetic energy required for cutting are produced by abrasive particles accelerated by pressurized water hitting the workpiece surface [4,5].

Almandine garnet, which is the most popular type of natural abrasive used in AWJM, is a reddish inert mineral composed of silicon dioxide (SiO_2_), iron III oxide (Fe_2_O_3_), and aluminium oxide (Al_2_O_3_), characterized by a Mohs hardness of approximately 8 and a specific mass of about 4 Mg/m^3^ [6,7]. The material has irregularly shaped grains with sharp or rounded edges. Although garnet is not regarded as toxic, exposure by inhalation, whether prolonged or repeated, may lead to respiratory and other health problems, for instance, eye irritation. Abrasives differ in mechanical and physical properties, e.g., particle size range or mass fraction, so they are selected depending on the process or equipment used.

Borkowski and Sokolowska [8] suggest that the pressure of water is responsible for the temperature in the cutting zone, which means that the higher the pressure, the higher the temperature. Researchers dealing with AWJM report that the workpiece temperature is relatively low, reaching up to 50 °C [9].

Most sources on AWJM also claim that there is no or negligible heat transfer during the process and that the workpiece material is not affected by the temperature as it is relatively low, ranging from 30 to 60 °C [5,10,11,12]. Some of the first researchers to suggest material structure changes due to temperature in AWJM include Imanaka et al. [13], who indicate that the workpiece temperature increases with increasing water jet pressure. For example, a study on polyvinyl chloride (PVC) [14] reports that the temperature in the jet impact zone may reach 175 °C. Another study [15] reveals that the temperature of Al 6061-T6 cut by AWJM, which was measured with thermocouples, does not exceed 75 °C. The research described in [16] shows that the temperatures of aluminium and steel cut by AWJM are not higher than 65 °C and 70 °C, respectively. However, from the investigations presented in [17], it is clear that in the AWJ cutting of steel, the temperature may exceed 450 °C.

The temperature in the cutting zone does not seem to affect the material structure when simple through cuts are made. For complex shapes, however, cutting involving large temperature changes is not desirable [1]. The modelling of the temperature field based on measurement data was undertaken by Ohadi and Cheng in [18]. It should be noted that thermal measurement in AWJM may not provide reliable results. Although it is naturally assumed that because of the highest temperature the greatest changes occur in the jet impact zone, thermal imaging does not confirm it. Thermographic cameras are able to detect only small temperature changes at the surface.

According to Arola and Ramulu [19], the heat transfer taking place in AWJM involves forced convection, with high energy needed to remove the workpiece material particles. Another important phenomenon is the friction of the abrasive water jet with the material surface. The rest of the heat generated in the process is dissipated. The structure of the material changes locally both in the jet impact zone as well as in the impact-affected zone; all these changes are induced by high temperatures [20].

Most of the energy produced by the abrasive water jet is used for cutting, which involves the erosion of particles [21]. Some of the energy is transferred to the material directly along the edge. From the energy balance analysis, it is evident that some of the kinetic energy is dissipated, causing plastic deformation (curved edges).The resulting heat generated locally in the workpiece leads to a local increase in temperature (very close to the cut edge).

As suggested by Kovacevic in [9], the temperature of the cutting medium increases when the water jet is formed in the nozzle. The higher the pressure of water in the jet, the higher the quality of the cut. When the pressurized water jet reaches the workpiece, kinetic energy is converted into thermal energy to be transferred to the workpiece material along the edge. The temperature in the jet impact zone is not easy to measure. Thermal imaging cameras can provide misleading results [22,23]. Very thick workpieces are particularly problematic; even in a direct measurement of temperature, readings are likely to be inaccurate.

Although the AWJM process is commonly referred to as one involving little or no heat transfer with no or negligible effect on the material structure [7], the latest studies [17] show that during the AWJM process, very high temperatures are reached, and these contribute to changes in the workpiece microstructure along the edge.

Phase transitions associated with high temperatures are responsible for higher stresses in the thin surface layer along the edge. As a result, new phases with different electrochemical properties, e.g., austenite, form. Their occurrence may result in changes in the material properties, especially the mechanical ones. Lower corrosion resistance, for instance, will lead to the worsening of the quality of a product and its shorter service life.

## 2. Materials and Methods

The investigations described in this article were conducted as part of international cooperation between the Kielce University of Technology, Poland and the VSB—Technical University of Ostrava, Czech Republic.

The abrasive water jet cutting of hot-rolled S235JR (1.0038) carbon steel with 0.19% carbon content was performed using a PTV WJ1020-1Z-EKO machine. During the process, the temperature was measured with a thermal imaging camera and NiCr-NiAl type thermocouples, able to register temperatures of up to 100 °C. The thermal imaging camera was a FLIR Systems Therma CAM SC640 featuring a 640 × 480 pixel focal plane array. It has a temperature range of −40 to +1500 °C and a thermal sensitivity of 30 mK at 30 °C. The camera with a field of view of 24° × 18°was placed at a distance of 0.3 m from a specimen. The measurements were taken at an image frequency of 120 Hz. The thermocouples used in the experiments were type K thermocouples by the Polish manufacturer CZAKI. The 0.5 mm thick wire with an insulated tip is able to withstand temperatures up to 1200 °C.

The chemical composition of S235JR steel determined through energy dispersive spectroscopy (EDS), given in Table 1, shows that the material complies with the requirements specified in standard EN 10025-2:2004. S235JR steel contains small amounts of chromium, nickel, and molybdenum to improve its hardenability [24].These elements also have the role of ferritizers.

The thermal conductivity of the material tested was low (58 W/m*K according to PN EN ISO 6946) [25] when compared to those of copper and silver (with the former being approx. 400 W/m*K and the latter equal to approx. 430 W/m*K) [22,23].

**Figure 1:**
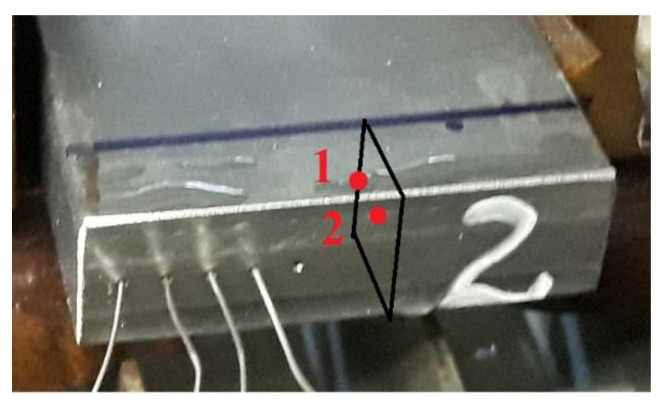
A specimen (i.e., Specimen No 2) with holes to measure temperature with thermocouples.

The specimens used for the experiments were prepared as shown in Figure 1. The holes in which the thermocouples were placed differed in depth so that the temperatures could be measured at different distances from the edge.

It was assumed that the temperature would be measured in the middle of the cross-section, where steady-state conditions are observed during cutting. The choice was based on the preliminary research and a review of the literature. The jet impact zone is where considerable deformation takes place, while the impact affected zone is characterized by the largest jet curvature, dependent, for example, on the cutting speed.

Figure 1 shows how the specimens were cut to analyse the material microstructure and phase composition. The black lines indicate the specimen area, and the red dots illustrate where the structure analysis was made.

The thermocouples were properly attached to prevent shifting during measurement, i.e., to ensure steady contact with the specimen surface.

The tests aimed to analyse how certain parameters and factors affected the abrasive water jet machining process. Table 2 shows the cutting parameters used in the experiments.

The composition and structure of the material after cutting were determined through computed tomography, optical microscopy, and X-ray diffraction analysis. The CT examinations were performed with a Nikon M2LES System at the Radiography and Computed Tomography Laboratory of the Kielce University of Technology. The microstructural analysis to study the morphology and the grain size of the material was conducted using a Nikon Eclipse MA200 (Minato-ku, Japan) optical microscope equipped with NIS 4.20-Elements Viewer imaging software. The phase composition analysis was carried out by means of a Bruker D8 Discover Plus combined with the Bruker Diffrac Suite software. Nanohardness was measured with an Anton Paar micro combi tester (MCT).

## 3. Results and Discussion

The temperature of the material cut by AWJM was measured using a thermal imaging camera and thermocouples. The measurements were taken under the conditions provided in Table 2.

The thermal imaging observations aimed to compare the temperature in the jet impact zone with that in the base metal. Although the actual temperature near the edge was higher, the readings did not exceed 30 °C. This suggests that the water jet prevented the camera from taking exact measurements as it covered the region where erosion occurred. The measurement errors were thus significant because the measurements were indirect and were affected by numerous factors. As the errors were also due to the ratio of the cut width to the area scanned by the camera, direct measurements were necessary.

Five holes with a diameter of 0.65 mm were drilled to depths of 4, 4.5, 5, 5.5, and 6 mm, respectively. The thermocouples used for this purpose were placed in four of them. The software used to register the temperature made it possible to select the sampling time, which was assumed to be 0.05 s. The accuracy of the temperature measurement was 0.1 °C.

**Figure 2:**
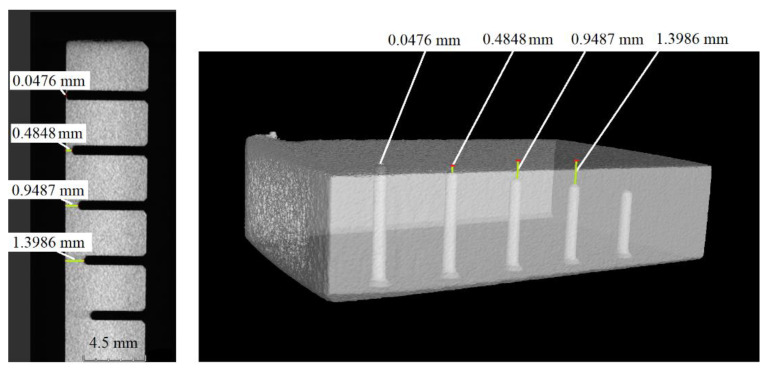
Locations of the thermocouples determined by VGStudio.

Computed tomography analysis was essential to determining the actual distances from the edge at which the thermocouples would be placed. The distance between the thermocouple measuring tip and the surface of the material cut was calculated as the thickness of the material after cutting. VGStudio 3.5.1 software helped visualize the distances from the edge, as illustrated in Figure 2.

**Figure 3:**
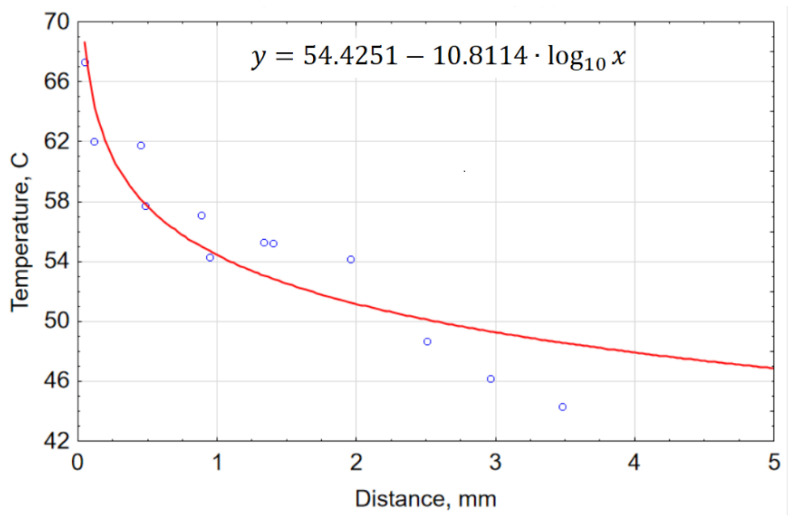
Temperature against distance from the edge in AWJM.

Only the maximum temperatures registered by the thermocouples were considered. The data were used to calculate the theoretical temperature at the edge. The plot in Figure 3 shows the relationship between the temperature and the distance from the edge.

From the results, it is clear that the maximum temperature registered by the thermocouples was 67.2 °C. It can thus be assumed that the research on AWJM claiming that the temperature does not exceed 100 °C is based on thermal imaging observations or surface measurements with thermocouples.

However, as described in [17], the authors indicate that the temperature in the jet impact zone may be higher than 0.4 times the melting temperature in the absolute temperature scale (K), which gives us approximately 450 °C. It should be noted that abrasive water jet machining requires high-density energy to make the particles erode so that the material can be split. The high pressure of the water jet combined with the small width of the cut is responsible for complex processes taking place in the cutting zone. Plastic deformation of grains is observed along the edge. In addition, some characteristic sparking can occur when the cutting is performed on steel containing niobium or zirconium.

The purpose of this study was to further investigate what temperatures can be reached during AWJM along the edge.

Since AWJM is a fast cutting process, it is important to take into account some specific properties of the material to be cut, such as thermal capacity or heat transfer coefficient. The energy delivered in the form of heat undergoes convection in all directions, including towards the measuring equipment. Surface temperature measurement with thermocouples does not seem effective in registering the actual temperatures in the cutting zone. The water jet not only cuts the material but also cools it afterwards. Temperature measurements with thermocouples prove that if too little energy is generated, it is immediately converted into heat. Most of the jet-induced energy produced during AWJM is used for the erosion of particles. The measurement results can also be affected by the inertia of the thermocouples. The high speed at which the cutting is carried out may be responsible for registering temperatures much lower than those occurring in the cutting zone.

**Figure 4:**
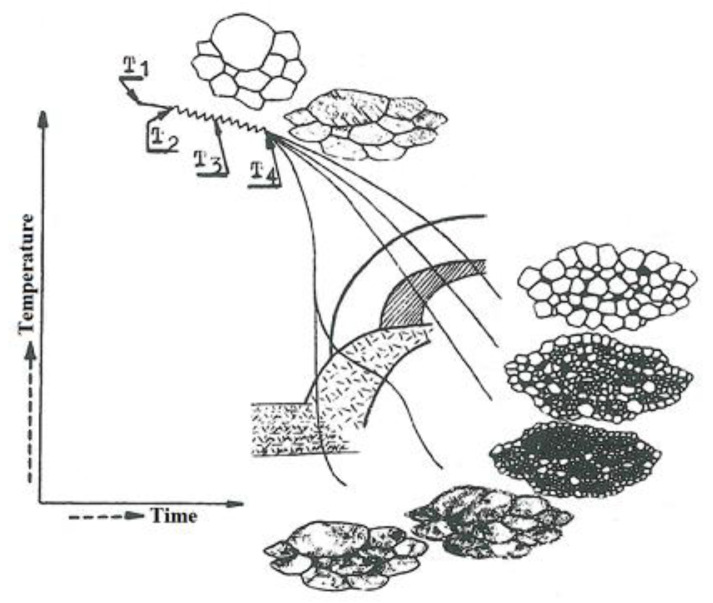
Influence of the cooling rate on the microstructure of steel in controlled rolling [26].

The phenomena caused by heat transfer in the jet impact zone resemble those observed during heat treatment processes. A properly selected heat treatment process ensures a desirable microstructure of a material, which strongly affects its mechanical properties. Hot metalworking can also contribute to microstructure changes. As indicated in [26], the type of microstructure depends on both the process temperature and the cooling rate. Figure 4 shows the types of microstructures of steel obtained in controlled rolling, according to the cooling rate. As can be seen from Figure 4, rapid cooling of steel is responsible for a coarse-grained microstructure, while at a lower cooling rate fine-grained sorbite or troostite occurs.

**Figure 5:**
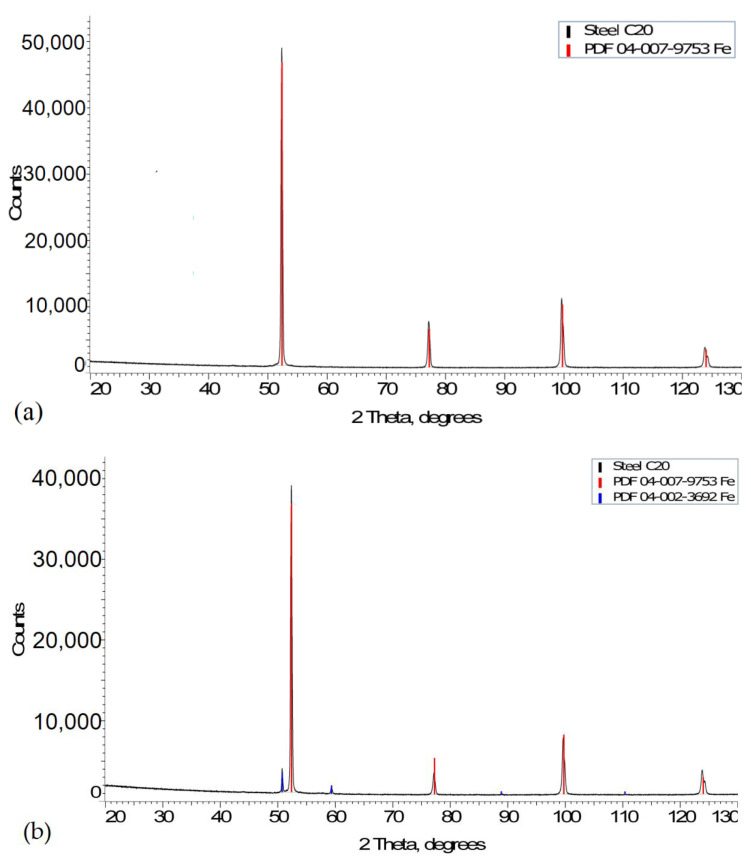
XRD patterns showing the phase composition: (**a**) base metal, (**b**) jet impact zone.

The time–temperature transformation (TTT) diagrams (Figure 4) were used to carry out an XRD analysis of the phase composition of the material along the edge and in the base metal zone. The X-ray diffraction analysis data were useful to determining the changes in the parameters of the crystallographic lattice, the size of subgrains (crystallites), and the phase composition. Figure 5 shows the diffraction patterns obtained through the qualitative XRD analysis to identify the phases present.

The results of the XRD analysis shown in Figure 5 provide evidence that two phases, Feα and Feγ, can be distinguished along the edge. In the base metal, only the Feα phase is reported. This suggests that eutectoid transformation took place, reaching a minimum temperature of 727 °C. The eutectoid temperature may be different under dynamic cutting conditions in AWJM. Figure 5b shows that the peaks representing austenite are much lower than the ferrite peaks but much higher than the background. The number of counts of the austenite quanta is sufficient; the peaks in blue above the background are easy to notice.

Another observation made during the experiments was a clear decrease in the number of medium-size crystallites in the plastic deformation zone when compared with that further from the edge. The size of Feα crystallites was calculated using the Williamson–Hall diagram:

- 61 nm for C20 steel close to the edge;
- 69 nm for C20 steel further from the edge.

**Figure 6:**
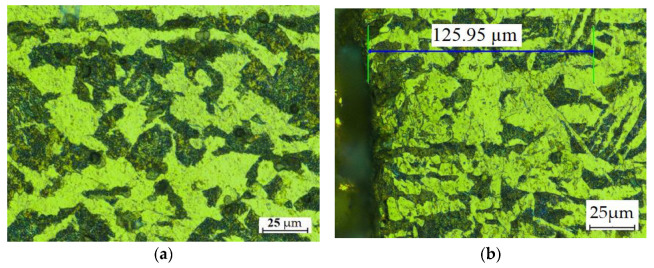
Microstructure of the hot rolled steel (**a**) before and (**b**) after the AWJM process.

The optical microscopy data confirmed the findings. Images of the steel microstructure are shown in Figure 6.

After the cutting, the material close to and along the edge had a fine-grained microstructure with smaller ferrite subgrains. A large number of such subgrains formed in the ferrite grains, as shown in Figure 6. The formation of the fine-grained structure, observed up to about 40 µm from the line of cut, was due to the occurrence of temperatures higher than the eutectoid temperature. After cutting with an abrasive water jet, the partially strand-like pattern of dark grains of pearlite and light grains of ferrite is no longer present. At larger distances, i.e., 50–150 µm from the line of cut, the average size of ferrite grains increases.

**Figure 7:**
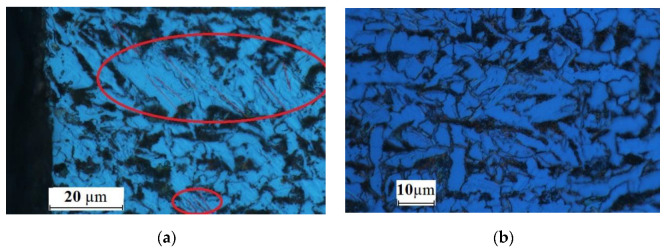
Polygonization in the microstructure of a metallographic specimen: (**a**) area close to the edge, (**b**) base metal.

**Figure 8:**
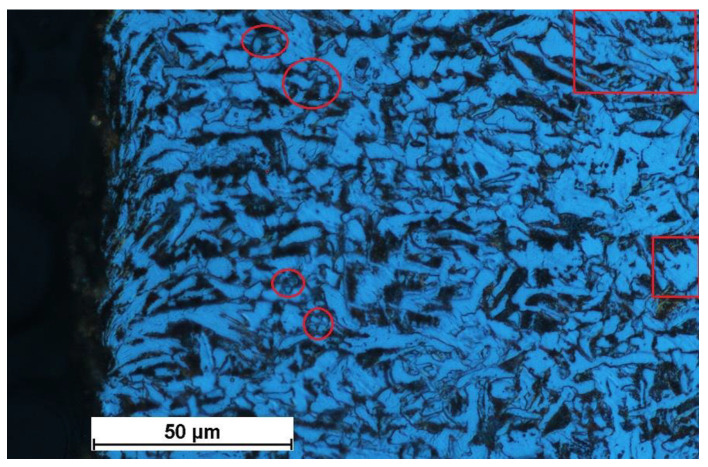
Recrystallization (red circles) and polygonization (red rectangles) in the microstructure of a metallographic specimen.

**Figure 9:**
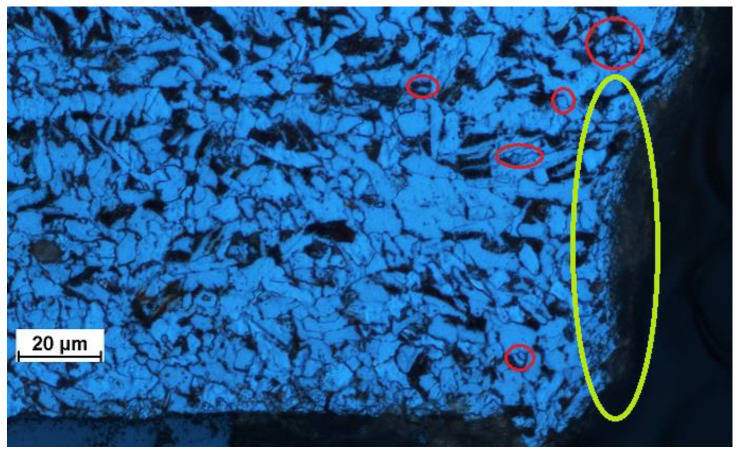
Recrystallization (red circles and yellow circles—at the cutting edge) in the microstructure of a metallographic specimen.

The fine-grained microstructure was due to plastic deformation and local heat transfer, both being a result of heat generated by erosion and friction. A considerable increase in temperature in the cutting zone causes certain changes in the workpiece material, including a higher density of grain sub-boundaries in the ferrite grains, where polygonization is also likely to occur (Figure 7 and Figure 8); a smaller average size of ferrite grains, indicating recrystallization (Figure 8 and Figure 9); and phase transition, causing the occurrence of the gamma phase (Figure 5), which is residual austenite. The austenite did not undergo transition, for instance, because of internal stress. Thus, the phase composition remained unchanged with time. In Figure 9, yellow was used to indicate the area where the material structure differed from that further from the edge. The area in the vicinity of the water jet is rapidly heated and then rapidly cooled. As the cooling rate is high, regions with bainitic or even martensitic structures are likely to occur. Further investigations are required to study the processes.

The heat transfer taking place at a sufficiently high temperature for a sufficiently long time causes the structure to become fine-grained in the whole volume. The effect is visible at close distances of up to 150 µm from the edge. The zone where the microstructure changes occur is narrow because the heating and cooling times are short. It can be assumed that when materials thicker than 15 mm are cut, changes may be observed at larger distances.

**Figure 10:**
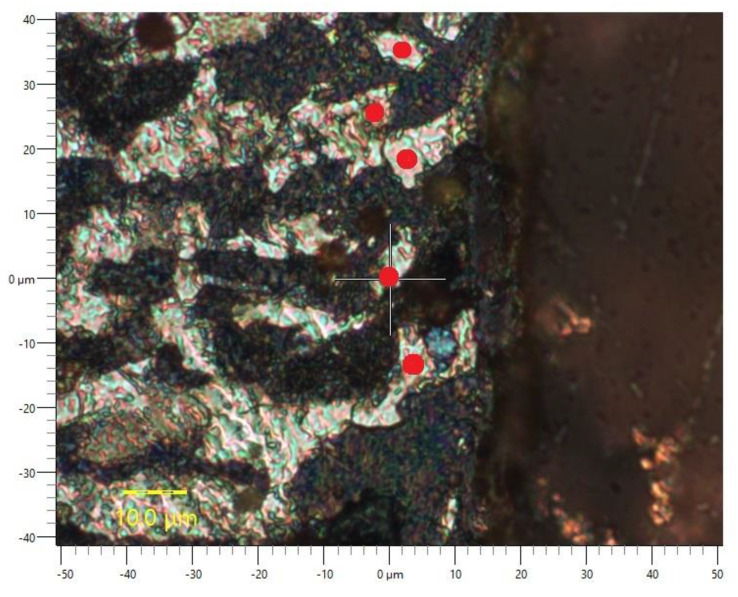
An OM image of the AWJ-cut edge with places where indentations were made.

Nanohardness measurements were conducted to determine the effect of the cutting temperature on the material hardness. The measurements were taken along the edge of the specimens cut and prepared for metallographic analysis by polishing and etching. Five measurements were performed for each specimen at a load of 10 mN. The red colour in Figure 10 indicates the places where the nanoindentations were made in the material.

The analysis of the light areas, which suggests the occurrence of ferrite or austenite, shows that the hardness there is higher than that of the base metal. The hardness in the jet impact zone reached 620.1 ± 12.2 HV, while that of the base metal was 423.9 ± 14.5 HV. The increase in hardness indicates an increase in the number of dislocations. This increase corresponds to an increase in the resistance of the material to indentation. The changes in hardness suggest heat transfer and plastic deformation. It can be concluded that eutectoid transformation must have occurred at a temperature higher than eutectoid temperature (727 °C for the equilibrium conditions).

The dark regions in Figure 9 indicate the occurrence of lower bainite or very fine upper bainite. Upper bainite has a needle-like structure with carbide precipitates, which dissipate light.

The hardness test results clearly show that in the jet impact zone the temperature is definitely higher than the eutectoid temperature (727 °C). Actually, temperatures may be higher locally, in a very narrow area along the edge (jet impact zone). Due to the inertia of the existing measuring devices, temperature readings cannot be very accurate as the changes are extremely fast. Further research is required to obtain more evidence of heat transfer involving locally high temperatures during the AWJM process.

The most exact method to determine the temperature in the gap in AWJM is by analysing the microstructures formed. Examinations of metallographic specimens provide information on heat transfer and other phenomena taking place during the AWJM process.

## 4. Conclusions

The research described in this article confirms that abrasive water jet cutting is a high-energy process resulting in locally high temperatures. The occurrence of locally increased temperature was confirmed through:

1. X-ray diffraction analysis, which indicated the gamma phase along the edge cut by AWJM;
2. Phase change analysis (the occurrence of austenite along the edge);
3. X-ray diffraction analysis, which showed smaller crystallites in the jet impact zone (61 nm) than in the base metal (69 nm);
4. Nanohardness measurements, which reported higher hardness of the material closer to the edge (620.1 ± 12.2 HV) compared with that further from the edge (423.9 ± 14.5 HV);
5. Microscopic observations, which showed changes such as polygonization and recrystallization.

The microscopic examinations confirmed the formation of a fine-grained microstructure along the edge. The significant decrease in the grain size was due to the thermal factor. This suggests that the temperature in the jet impact zone definitely exceeded the recrystallization and eutectoid temperature. As rapid changes in temperature and considerable deformation were reported along the edge, it is clear that the temperature was different from that specified for the equilibrium conditions.

It can thus be concluded that during AWJM the temperature is higher than the eutectoid temperature. With the equipment available these days, however, it is impossible to determine its exact value. Further research is essential to find more evidence on the occurrence of locally increased temperature. The higher density of subgrains and grain sub-boundaries indicates that processes of polygonization or recrystallization took place in the material.

## Figures and Tables

**Table 1:** Chemical composition of the steel tested (wt. %).

	C, %	Mn, %	Cu, %	P, %	Si, %	S, %	Other Elements, %
EN 10025-2:2004 requirements	max. 0.19	max. 1.50	max. 0.60	max. 0.045	-	max. 0.045	-
Material tested	0.19	1.38	0.073	0.024	0.01	0.009	0.039 Ni 0.055 Cr 0.009 Mo

**Table 2:** Parameters and factors affecting the AWJM process.

Variable	Value
Pump pressure	380 MPa
Nozzle orifice diameter	0.25 mm
Mixing tube diameter	1.02 mm
Mixing tube length	76 mm
Abrasive mass flow rate	250 g/min
Abrasive type	Australian garnet #80
Standoff distance	2 mm
Cutting speed	50 mm/min

## Data Availability

No publicly archived datasets are reported or used.
